# Land Ice Freshwater Budget of the Arctic and North Atlantic Oceans: 1. Data, Methods, and Results

**DOI:** 10.1002/2017JC013605

**Published:** 2018-03-05

**Authors:** J. L. Bamber, A. J. Tedstone, M. D. King, I. M. Howat, E. M. Enderlin, M. R. van den Broeke, B. Noel

**Affiliations:** ^1^ School of Geographical Sciences University of Bristol Bristol UK; ^2^ Byrd Polar Research Center Ohio State University Columbus OH USA; ^3^ School of Earth and Climate Sciences University of Maine Orono ME USA; ^4^ Institute for Marine and Atmospheric Research Utrecht University Utrecht Netherlands

**Keywords:** Arctic, North Atlantic, freshwater budget

## Abstract

The freshwater budget of the Arctic and sub‐polar North Atlantic Oceans has been changing due, primarily, to increased river runoff, declining sea ice and enhanced melting of Arctic land ice. Since the mid‐1990s this latter component has experienced a pronounced increase. We use a combination of satellite observations of glacier flow speed and regional climate modeling to reconstruct the land ice freshwater flux from the Greenland ice sheet and Arctic glaciers and ice caps for the period 1958–2016. The cumulative freshwater flux anomaly exceeded 6,300 ± 316 km^3^ by 2016. This is roughly twice the estimate of a previous analysis that did not include glaciers and ice caps outside of Greenland and which extended only to 2010. From 2010 onward, the total freshwater flux is about 1,300 km^3^/yr, equivalent to 0.04 Sv, which is roughly 40% of the estimated total runoff to the Arctic for the same time period. Not all of this flux will reach areas of deep convection or Arctic and Sub‐Arctic seas. We note, however, that the largest freshwater flux anomalies, grouped by ocean basin, are located in Baffin Bay and Davis Strait. The land ice freshwater flux displays a strong seasonal cycle with summer time values typically around five times larger than the annual mean. This will be important for understanding the impact of these fluxes on fjord circulation, stratification, and the biogeochemistry of, and nutrient delivery to, coastal waters.

## Key Points

- Freshwater has been accumulating in Baffin Bay for 20 years
- The freshwater anomaly has reached 6,300 km^3^
- Important source of labile nutrients to coastal water

## 1 Introduction

Observational and modeling studies indicate that the freshwater budget of the Arctic and sub‐Arctic North Atlantic (SNA) oceans have been changing over the last few decades (Carmack et al., 2016; Prowse et al., 2015; Swift et al., 2005) with possible consequences for the large‐scale circulation of the region (Proshutinsky et al., 2015) and stratification of the upper ocean (Carmack et al., 2016). Ocean GCMs are generally forced by reanalysis products that include continental river runoff but no component due to land ice mass changes (Griffies et al., 2009). This is, in part, due to relatively poor constraints on this component in the past and, in part, due to practical difficulties in including a freshwater flux (FWF) that is not directly or indirectly computed by the reanalysis model. To date, land ice has not been an interactive component in Earth System Models, further hampering efforts to include it in ocean reanalyses. It could also be argued that the contribution from land ice to the regional ocean freshwater budget has been relatively small compared to, for example, inter‐annual variability in precipitation minus evaporation (P‐E) (Peterson et al., 2006). For the Arctic and SNA Oceans this is no longer the case, as we demonstrate here. Mass loss from the Greenland Ice Sheet (GrIS) has been increasing since the mid‐1990s (van den Broeke et al., 2009) and for the largest ice caps in the Canadian Arctic Archipelago (CAA) since the mid‐2000s (Gardner et al., 2011). This mass loss is controlled by two processes: surface mass balance (SMB) and ice discharge. The former comprises gains from snowfall (accumulation) and losses from melting (runoff). SMB reacts quasi‐instantaneously to changes in surface energy balance, such as temperature and incoming solar radiation, and precipitation while discharge has a more complex, and delayed, response to changing atmospheric and oceanic conditions (Moon et al., 2012). In this study, we examine the role of both components and their temporal trends (i.e., runoff and discharge). However, we only consider changes in runoff in the SMB as this is the only component of the SMB that directly influences the FWF. In this context, it is important to note that a glacier or ice sheet can be gaining mass through increased snowfall while concurrently contributing to an increased FWF via enhanced melting and runoff. Thus, ice mass balance and FWF trends are not necessarily synonymous and an increase/decrease in one may not be matched be an equivalent trend in the other.

A 20^th^ Century reconstruction of the SMB of the GrIS (1900–2015) indicates relatively modest variability in runoff, with a decadal increase during the well‐documented period of relative warmth during the 1930s and relative stability from 1940 to 1995 (Fettweis et al., 2017). Since 1995, the ice sheet has experienced an unprecedented increase in runoff that exceeds any other period during the last 115 years in both absolute magnitude and amplitude of the change (Fettweis et al., 2017). The increase in runoff also approximately coincided with an increase in discharge (Fettweis et al., 2017; Rignot et al., 2008). This mass loss constitutes a cumulative FWF anomaly close to areas of overturning circulation in the SNA, and has been increasing roughly monotonically since 1995 (Bamber et al., 2012). It is noteworthy that, over the same time scale, the Arctic has experienced an atypical period of anticyclonic circulation, which has been associated with changes in the freshwater balance of the Arctic and SNA Oceans (Proshutinsky et al., 2015). Hence, while the freshwater anomalies are relatively small in terms of their absolute flux (O(10) mSv) they may play a central role in the changing climate of the region.

The GrIS is the largest ice mass in the Northern Hemisphere by two orders of magnitude and is currently the single greatest source of barystatic sea level rise (Vaughan et al., 2013). Numerous freshwater forcing experiments have been conducted to investigate the potential impact of increased fluxes from the GrIS. Many of the earlier studies have used an idealized FWF, typically averaged zonally and sometimes with unrealistically large flux anomalies (Gerdes et al., 2006; Hu et al., 2009; Jungclaus et al., 2006). More recently, experiments have used an observational and model‐based estimate of FWF from the GrIS to investigate its potential role on the hydrography of the SNA and influence on the Atlantic Meridional Overturning Circulation (AMOC) (Boning et al., 2016; Dukhovskoy et al., 2016; Gillard et al., 2016; Marsh et al., 2010; Yang et al., 2016). From these analyses, it has been suggested, for example, that changes to deep Labrador Sea Water are linked to enhanced FWF from the GrIS (Yang et al., 2016) and that, if the FWF trend continues, it could play an important role in modulating the strength of the AMOC (Boning et al., 2016). These studies have considered the contribution from the GrIS alone or have not had sufficient resolution to determine where the freshwater enters the ocean, whether it is in solid (icebergs) or liquid (surface and subglacial melt) phase, or its seasonal cycle. Ocean GCMs, in general, cannot handle solid ice fluxes (icebergs) in a physically realistic manner and have included them as part of the liquid phase at their source. This may misrepresent their role in modifying water mass properties as icebergs can drift thousands of kilometers from their original source (Marsh et al., 2015). Recent work with the Nucleus for European Modelling of the Ocean (NEMO) framework aims to specifically account for iceberg processes (Marsh et al., 2015) and has been extended for use in the Southern Ocean (Merino et al., 2016). Thus, ocean GCMs are beginning to be developed that can incorporate both liquid and solid FWF phases and with sufficient resolution to be eddy resolving, which is important for determining the trajectory and fate of the freshwater (Boning et al., 2016).

Here, we combine high resolution (in both time and space) satellite observations of solid ice discharge (D), with regional climate model output of surface runoff (R) to determine the contribution of land ice to the changing freshwater balance of the Arctic and SNA oceans for the period 1958–2016. We partition the FWF between solid and liquid phases and resolve the sources at the individual catchment and glacier scale at monthly time steps. Such information is not only important for the large scale hydrography of the surrounding oceans but also for modeling and understanding fjord circulation, ice/ocean interactions (Straneo & Heimbach, 2013; Straneo et al., 2011) and impacts on biological productivity in near coastal waters (Bhatia et al., 2013; Hood et al., 2009). In this paper, we present the data, methods and results, while a companion paper explores the implications for the hydrography of the surrounding seas and potential oceanographic impacts.

## 2 Methods and Data Sets

As mentioned above, the total FWF comprises a liquid component, made up of runoff from land ice and snowmelt on tundra (R) and a solid component (D), here defined as the flux of ice crossing the grounding line of marine‐terminating glaciers around the margins of the GrIS. In sections 2.1 and 2.2 we describe, in detail, how these two components were determined. In summary, we estimate the solid ice fluxes for the GrIS using satellite‐based observations of surface velocity alongside a new compilation of ice thickness (Morlighem et al., 2017). We have a sub‐annual time series of velocities for 2000–2016 and annual values for 1992–2010, 1958, and 1964. For those years with no observations, we employ a correlation between time‐averaged R and D. To estimate R, we use the output of a regional climate model (RACMO2.3p2) that has been coupled to a snow diagenesis model, forced by atmospheric re‐analyses (ERA‐40 and ERA‐Interim) for the period 1958–2016 (Noel et al., 2017). The runoff is routed to coastal points and combined with the discharge to produce a complete FWF time series along the margins of the land areas included in the study.

### 2.1 Greenland Solid Ice Discharge

Solid ice discharge (D) is the product of surface velocity and ice thickness along an outlet glacier flux gate, typically located near and upstream of the grounding line: the junction between the grounded ice sheet and the ocean. Thickness data are generally obtained from ice penetrating radar data measurements, while velocities are derived from synthetic aperture radar interferometry (Rignot et al., 2008) (henceforward R2008) and feature tracking of visible and radar satellite imagery (Enderlin et al., 2014). Here, we attain high temporal and spatial coverage by supplementing and extending R2008 with a new data set that captures surface velocities at sub‐annual temporal resolution for 195 outlet glaciers across Greenland. This data set was developed by co‐authors King and Howat and is henceforward called K&H2017. These two data sets were combined to produce ice sheet wide discharge estimates with monthly time steps. Due to gaps in sampling, interpolation was required in time. We also corrected for biases between K&H2017 and R2008. In a comparison of these data sets, we identified an approximate 100 km^3^ difference in their annual estimates of D for the same epoch (Yang et al., 2016). Comparison with independent mass balance data from satellite altimetry and GRACE indicated that there was a bias in the magnitude of the R2008 discharge estimates, which we have corrected for in this new analysis, possibly due to limits in the quality of the ice thickness data available when R2008 was produced. As the multiyear trends in D are very similar in both estimates, this correction only affects the absolute magnitude of D in R2008 rather than the anomaly.

First, we describe the approach used to derive the seasonally varying discharge for the 195 outlet glaciers, which has not been previously presented. Monthly glacier discharge values were estimated by first deriving continuous time series of outlet glacier velocities. Most velocity estimates were obtained from feature‐tracking techniques applied to combinations of both repeat‐pass and cross‐path orthorectified imagery obtained from ASTER, LANDSAT‐7 and LANDSAT‐8. Existing velocity data products from RADARSAT‐1 and −2, and TerraSAR‐X satellites were also included when available.

When SAR products are unavailable, data gaps during winter are common due to the limitations of optical imagery at high latitudes. To overcome this, data were interpolated to fill monthly gaps using a glacier‐specific seasonality, constructed from available velocity data for a particular month, available throughout a representative sample of the time series.

**Figure 1:**
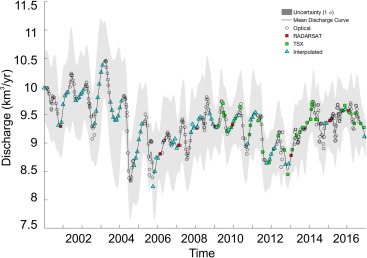
Example of continuous discharge time series for Store Glacier, Greenland (black line). Estimates extracted from optical imagery are shown as black circles, with additional interpolated estimates shown as blue triangles. Estimates calculated using radar observations are shown as red squares (RADARSAT −1 and −2), and green squares (Terra‐SAR‐X). The uncertainty (1 σ) accompanying the continuous discharge curve is depicted by gray shading.

A Monte Carlo simulation technique was then used to estimate a continuous discharge curve from the dense time series. The simulation, performed for 1,000 iterations at each individual glacier, fits a smooth cubic spline function for each time series along individual outlet glacier flux gates. On each iteration, the spline function is fit to a new set of perturbed values randomly selected assuming a normal distribution of error. The continuous curve is then sampled at daily intervals, with errors being found by taking the standard deviation of the curve estimates generated from the 1,000 iterations at each point in time. An example of a final discharge time series for Store Glacier, western Greenland is shown in Figure 1. The monthly data are averages of the daily discharge data and have been extended to the end of 2016 (compared to 2009 in Bamber et al., 2012) This data set constitutes K&H2017.The two discharge data sets have different spatial and temporal sampling characteristics, as well as covering different time periods. We combined them to produce monthly estimates of D for the 195 outlets in K&H2017, which provides monthly estimates of D for 2000–2016. Meanwhile, R2008 (updated in Rignot & Mouginot, 2012) derived mean annual estimates of discharge in regional drainage basins encompassing ∼95% of the ice sheet for 1958, 1964, and 1992–2009. To combine these two data sets, we first placed each of the K&H2017 outlet glaciers into a drainage basin defined by R2008 and then calculated basin‐wide K&H2017 totals. This analysis revealed that of the 100 km^3^/yr discrepancy, about 40 km^3^ is attributable to outlets along the eastern margin between 61.8 and 65.2 ^o^N, and ∼27 km^3^ is attributable to outlets on the south and south‐west margin between 61.5 and 64.8 ^o^N. The remaining differences do not show any significant spatial clustering. Next, we calculated a scaling factor between R2008 and K&H2017 mean annual D totals for each drainage basin for the overlapping period of 2000–2009, and applied these scaling factors to R2008 to adjust for the aforementioned bias in R2008. Next, we used K&H2017 to estimate the mean annual proportion of discharge that each outlet glacier drained from their respective drainage basin. We used these estimates to split the R2008 drainage basin mean values for 1958, 1964, and 1992–1999 into outlet glacier contributions for these years. From 2000 onward, we used K&H2017, except in basins without an estimate, where we used annual, unscaled R2008 measurements of discharge.

**Figure 2:**
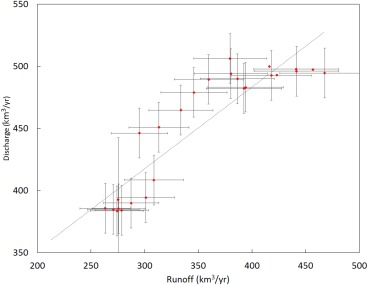
Five year running mean Greenland ice sheet runoff versus annual discharge estimates for 1962, 1992–2012, with the least squares linear trendline shown as the dotted line.

We estimated the mean monthly discharge to obtain the amplitude of the seasonal cycle at each outlet, which we then applied to annual estimates of discharge at these outlets prior to 2000 (in other words we assumed the seasonal cycle is time invariant). This provided a monthly time series for the whole ice sheet for 1958, 1964, and 1992–2016. For those years with no observed discharge value we followed the approach in (Bamber et al., 2012) by fitting a linear least squares regression between ice‐sheet‐wide runoff over the previous four years and the current year with ice‐sheet‐wide D for that year (correlation coefficient of 0.81, p < 0.01). The results of this linear regression are shown in Figure 2. We excluded 1958 in the regression as the discharge data for this year has a larger, but poorly defined, uncertainty (Rignot et al., 2008). We distributed these ice‐sheet‐wide D totals to individual outlet glaciers according to the mean percentage contribution of each outlet to ice‐sheet‐wide D during 2000–2016.

Errors in discharge, where measured ice thickness data are available, are about 2% and 6% where thickness is interpolated (Enderlin et al., 2014). We have, however, had to combine two different data sets that cover different epochs and glaciers. To account for the additional uncertainty introduced by this interpolation, we conservatively estimate a 6% error on discharge for years when it was observed (1992–2016) (Bamber et al., 2012). For those years where we use the relationship illustrated in Figure 2, we take the RMS misfit of the regression (20.6 km^3^/yr) added to the uncertainty on an individual measurement as the error estimate, which equates to about 11% of the mean D value for the interpolated years (1959–1963; 1965–1991).

While the analysis above provides a time series of D for the GrIS, we have not considered discharge variability for glaciers and ice caps elsewhere. For the CAA, we found that the surface mass balance from the regional climate model (discussed in section 2.2) matched the mass loss observed by the GRACE satellites for the same epoch. For the period 2004–2009, Gardner et al. (2011) estimate that 8% of the increased mass loss was from D. This is equivalent to about 5 km^3^/yr and, therefore, small compared to other fluxes. For the CAA we believe, therefore, that changes in D are unimportant (Millan et al., 2017), while for Iceland there are no marine terminating glaciers. For Svalbard, surface mass balance only accounts for 2 km^3^/yr of mass imbalance compared with 7 km^3^/yr mass loss obtained from GRACE for 2003–2015. The difference of 5 km^−3^/yr is, however, just under 2% of the total FWF anomaly for the same time period and comparable to the contribution from the CAA. We consider, therefore, that ignoring changes in D outside of Greenland introduces a small underestimate of the land ice FWF anomaly of about 10 km^3^/yr.

### 2.2 Land Ice and Tundra Runoff

To estimate runoff from the GrIS and GIC we used the output of a regional climate model (RACMO2.3p2 for the GrIS and version p1 for GIC), which has been statistically downscaled to 1 km from the native resolution of the model of 11 km (Noel et al., 2015; Noël et al., 2017). Monthly data from 1958 to 2016 at 1 km nominal resolution were projected onto a 5 km polar stereographic grid. We applied a latitudinal scaling correction to preserve area and, hence, volume, away from the projection central meridian (Snyder, 1987). To determine runoff fluxes along the coast, we routed runoff from its source to the downstream coastal point. First, for Greenland we split runoff into its ice and tundra source components using the land type classification employed in the regional climate model. We then used the PyGeoProcessing library (http://pythonhosted.org/pygeoprocessing/) in conjunction with a digital elevation model (Howat et al., 2014) of the land (tundra component) and ice surface (ice sheet component) to route the water to the coastal margin. We allocated each coastal pixel to an oceanographic sub‐basin as defined by the International Hydrographic Organisation (IHO, 1953). Conservation of mass (runoff) was assessed by comparing the total flux at the ocean boundary with the integral over the ice and tundra areas to ensure that none was lost during the routing procedure or between the ice edge and the closest ocean grid cell. Although RACMO2.3p1 contains improved physics compared to the version used in the previous FWF assessment, we assume the same errors as given in Bamber et al. (2012), namely 20% for land ice runoff and 10% for tundra runoff. We assume that the errors in runoff are systematic and therefore correlated between different ice masses but not with observed values of D.

## 3 Results

In the following sections, we consider terms that are defined below:

(1) $${FWF}_{T}=D+R_{GRIS}+R_{t}+R_{GIC}$$

Where *FWF_T_* is the total FWF from all components, *D* is solid ice discharge in Greenland, *R_GRIS_* is Greenland ice sheet runoff, *R_t_* is tundra runoff on non‐glaciated Greenland land and *R_GIC_* is glacier and ice cap runoff outside of Greenland. Here, this latter term comprises the CAA, Svalbard and Iceland (cf. Figure 4), which are the relevant land ice sectors for the freshwater budget of the SNA. The Russian Arctic was not included as regional climate model output was not available for this sector. The ice caps in this region are estimated to have been losing mass at a rate of 11 ± 4 Gt yr^−1^ between 2003 and 2009 (Gardner et al., 2011). This is about 3% of the mass imbalance of the land ice included in this study for the same period. *R_t_* shows no trend over time and we do not see a trend in P‐E over the CAA either. The tundra runoff term is, therefore, part of the mean FWF from land. As discussed later, some ocean reanalyses include a P‐E term for Arctic land areas and some do not. We only discuss this term for Greenland. It is provided as a separate field and can, therefore, be included or excluded as necessary for both Greenland and the other land masses shown in Figure 4.

(2) $${FWF}_{A}={FWF}_{T}-reference\;FWF$$

Where *FWF_A_* is the FWF anomaly and the reference FWF is the mean of years 1960–1990.

(3) $${FWF}_{CA}=\;\int_{1958}^{yr\;}{FWF}_{A}\;dt$$

**Figure 3:**
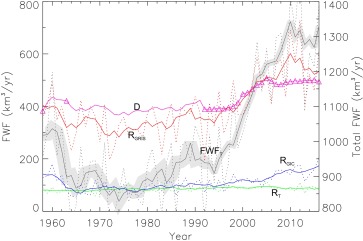
FWF components from the GrIS and GIC in km^3^/yr. For reference, 1 mSv is equivalent to 31.6 km^3^/yr. Solid lines are 5‐year moving averages, dashed lines are the mean annual values. Triangles for D (magenta), show the years where values were observed. For other years (1959–1961, 1963–1991) they were estimated from the relationship shown in Figure 2. The total FWF from all sources is shown by the solid black line plotted against the right‐hand Y axis. The 1 σ uncertainty in the total FWF is shown by the gray shading.

Where *FWF_CA_* is the cumulative anomaly. The time series for the five FWF components defined in equation (1) are shown in Figure 3.

The surface runoff components of the FWF show relatively large inter‐annual variability, driven by the common underlying variability in the climate forcing, primarily near‐surface air temperature, solar radiation and to a lesser extent precipitation. Discharge, integrated over the ice sheet, varies more smoothly in time as this term is controlled by internal ice sheet processes that have a longer response time to external forcing. In addition, it is, by default, smoothly varying for the years where it was interpolated using five years of runoff data (section 2.1). The detrended RMS inter‐annual variability for *FWF_T_* is 89 km^3^/yr, which is considerably less than the increase seen since 1995. Between 1995 and 2016, the *FWF_T_* increased by about 400 km^3^ (equivalent to 12.6 mSv). This absolute difference in FWF is comparable to, for example, the five‐year variability in P‐E over sub‐polar basins (Peterson et al., 2006) but taking place over a longer time scale (∼20 years). The increasing trend in FWF peaks in 2012, associated with the anomalous melt event over Greenland in the same year (Nghiem et al., 2012). The values for 2013–2016 (1,177 km^3^/yr), while less, remain significantly above the mean for the 59 year period studied (972 km^3^/yr). As noted in the introduction, a reconstruction of the 20^th^ Century surface mass balance for the GrIS, indicates that recent runoff fluxes (since the mid‐2000s) are unprecedented for the last 115 years and their acceleration since 1995 is also unprecedented since at least 1900 (Fettweis et al., 2017).

**Figure 4:**
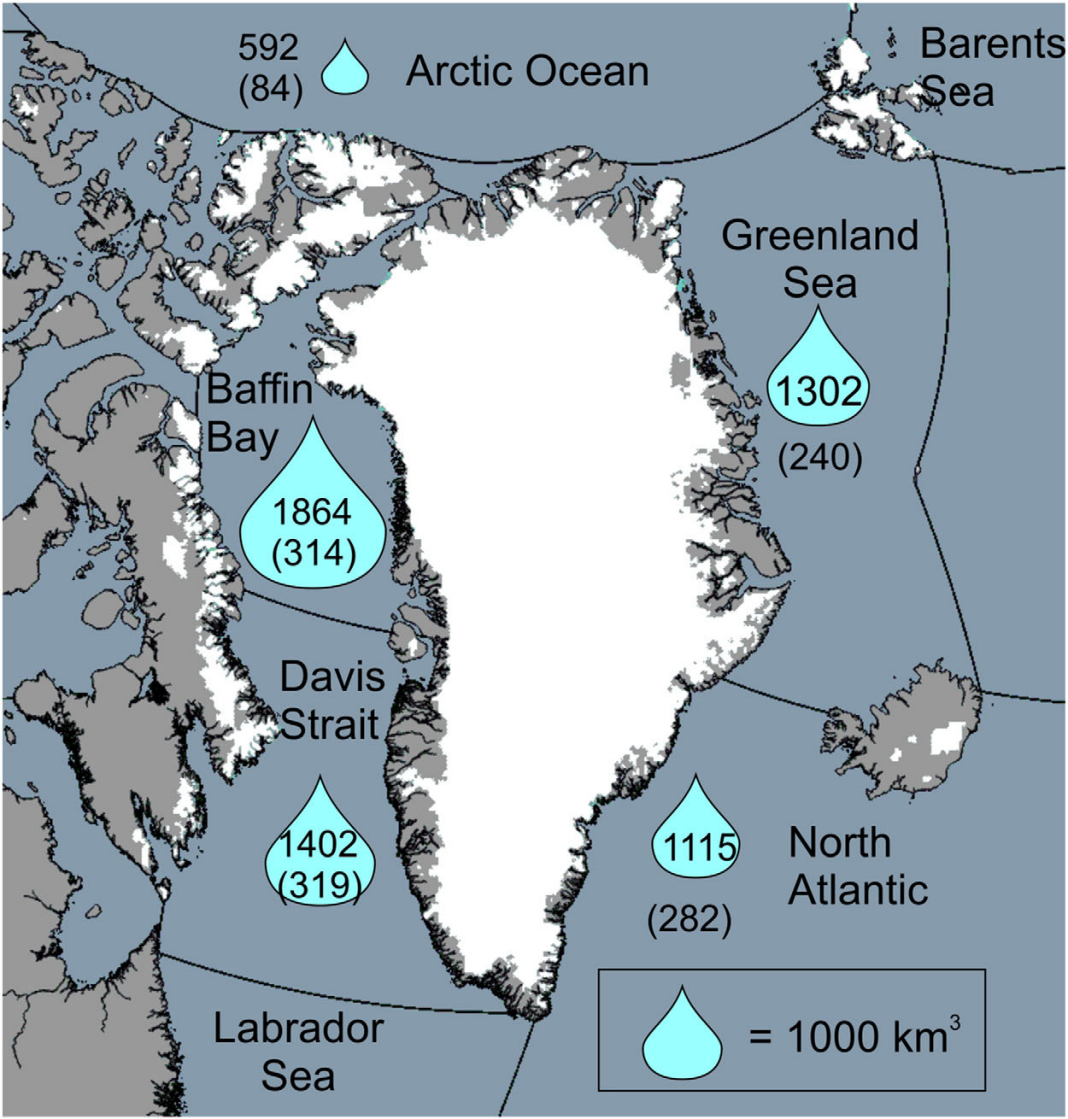
The IHO defined ocean basins used to partition the land ice FWF originating from the GrIS and GIC. All the land ice contributions included are shown in white. Non glaciated areas are shown in grey. The only runoff from a non glaciated area included in the FWF analysis is from the tundra in Greenland (R_T_). However, no trend for R_T_ exists so it does not affect the anomalies. The numbers refer to the cumulative anomalies (FWF_CA_) in km^3^ for each ocean basin using the period 1960–1990 as reference. The blue “droplets” are scaled by the size of the cumulative anomaly. The rates (km^3^ yr^−1^) are shown in Figure 3 and the time series in Figure 5. The numeric values in brackets are the decadal mean FWF for each basin for the period 2007–2016, discussed in section 5.

These changes over Greenland were driven by regional climate that largely also affects other parts of the Arctic: the large scale trends for the CAA are reasonably well correlated (Figure 3). Thus *FWF_CA_* since 1995 will also be unprecedented for at least the last century and it is this quantity (i.e., the anomaly) that is of more relevance to the hydrography of the Arctic and SNA oceans rather than the absolute change in FWF. To examine the cumulative anomalies, we partitioned the FWF into ocean basins as defined by the International Hydrographic Organization (IHO) (IHO, 1953) using their shapefile data (downloaded from http://www.marineregions.org in December 2016). The domain and basins are shown in Figure 4, which also indicates the total cumulative anomaly for each basin. Years 1960–1990 were used as the reference period for the anomalies (cf. equation (2)) as, during this time, GrIS runoff was relatively stable (cf. Figure 3) and the ice sheet was close to balance (Fettweis et al., 2017; van den Broeke et al., 2016), although likely not in equilibrium (Kjeldsen et al., 2015). The largest cumulative anomaly is now found in Baffin Bay and originates from both increased runoff from the GrIS and the northern CAA (Figure 3). This is in contrast to the previous assessment, where the period up to 2010 only was covered, and where it was the North Atlantic sector that experienced the largest *FWF_CA_* (Bamber et al., 2012).

**Figure 5:**
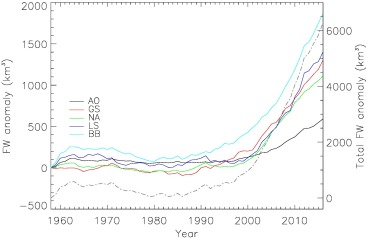
Cumulative FWF anomalies from Arctic land ice sources partitioned by ocean basin. AO = Arctic Ocean, GS = Greenland Seas, NA = North Atlantic, LS = Labrador Sea, BB = Baffin Bay. Ocean basins are indicated in Fig 4. The reference period for the anomalies is 1960–1990. The dashed line shows the total anomaly for all basins, using the right‐hand Y axis.

The time series of *FWF_CA_* by ocean basin, alongside the total for all basins, is shown in Figure 5. A small contribution from Svalbard and Iceland enters the Barents Sea basin (mean value 5 km^3^/yr), which we do not discuss further. Not surprisingly, anomalies begin to increase during the mid‐1990s when runoff over Greenland also shows a marked acceleration (Fettweis et al., 2017). There are, however, some regional differences in both timing and rate of change in *FWF_CA_*, with the largest and earliest trend occurring in Baffin Bay. Positive anomalies have been accumulating for about the last two decades and although the gradient reduced after 2012, the anomalies are still increasing relative to the 1960–1990 mean. Regional climate model projections suggest that the *FWF_A_* for Greenland, and by implication the CAA, will continue to increase for at least the next century (Fettweis et al., 2013). It is also, notable, that the total cumulative anomaly from all sources considered here is 6,300 ± 316 km^3^, which is about twice the value reported previously (Bamber et al., 2012). This is a result of extending the time series to 2016 and the inclusion of glaciers and ice caps outside of Greenland. As a consequence, the largest *FWF_CA_* is found for Baffin Bay, close to the CAA, which is responsible for about 9% of the anomaly for this basin. Observational evidence for the existence of a positive freshwater anomaly for this basin comes from satellite and *in‐situ* observations (Carret et al., 2017) and is also evident in the ORAP5 ocean re‐analysis product (Zuo et al., 2015) for at least the period 2003–2010. This freshening has been associated with land ice melting (Carret et al., 2017). The mean *FWF_A_* since 2000 in Baffin Bay is 90 km^3^/yr (3 mSV).

Four factors are responsible for the differences in the magnitude and characteristics of our new time series when compared to the previous analysis (Bamber et al., 2012). First, we use a new and updated time series for D, which both captures the seasonal cycle and provides much higher sampling in time of all the glaciers incorporated. The absolute discharge flux has been reduced by about 100 km^3^/yr although the accelaration since the 1990s is similar. Second, the regional climate model output has been downscaled to 1 km, which provides improved resolution of, in particular, peripheral glaciers and ice caps (PGIC) around the margins of Greenland that are not directly connected to the ice sheet (Bolch et al., 2013), and also the ablation zone of the ice sheet. It is predominantly melt from these peripheral glaciers that has resulted in higher runoff rates from the island of Greenland as a whole compared to the previous study. As a consequence of these two improvements, runoff exceeds discharge, for the first time, after about 2008 (Figure 3). Third, we have included GIC outside of Greenland (R_GIC_), which contribute a mean FWF of 109 km^3^/yr over the 59 years of our data but, more importantly, show a marked and steady increase, predominantly into Baffin Bay, from the early 2000s. Fourth, and most importantly for the cumulative anomaly, we have extended the time series from 2010 to the end of 2016. This increases *FWF_CA_* by about 2,000 km^3^ (Figure 5). While the five‐year smoothed FWF peaked in 2010 (Figure 3), and does not appear to be continuing to accelerate, it is nonetheless about 400 km^3^/yr (13 mSv) higher than during the early 1990s and still contributing to a positive FWF anomaly to all ocean basins.

## 4 Discussion and Conclusions

A thorough review of the role that freshwater fluxes play on physical and biogeochemical properties of the Arctic Ocean and sub Arctic basins can be found in (Carmack et al., 2016). We note, however, that assessments of the freshwater budget of Arctic seas, including Baffin Bay and the Davis Strait, appear to have significantly underestimated the contribution of land ice. In a recent, comprehensive FW budget analysis, land ice was estimated to contribute 370 km^3^/yr for 2000–2010 (Haine et al., 2015) based on (Bamber et al., 2012). The ocean basins defined in our study are not identical to the domain used in (Haine et al., 2015) and a direct comparison is, therefore, difficult. Nonetheless, we note that their domain includes all of Baffin Bay and the Arctic Ocean and approximately a quarter of the Greenland Sea and Davis Strait basins illustrated in Figure 4. Using these areas as a rough guide, we obtain a mean FWF of 514 km^3^/yr for the same time period, increasing to 563 km^3^/yr for 2007–2016, which is 52% larger than (Haine et al., 2015) as a consequence of the increased melting in recent years plus the inclusion of glaciers and ice caps.

Several studies, using ocean GCMs, have explored the potential impact of GrIS melting on the hydrography of the SNA and the strength of the AMOC (Boning et al., 2016; Gillard et al., 2016; Yang et al., 2016). They have, however, relied on incomplete land ice FWF estimates and, as a result, have generally forced their models with smaller anomalies and overall trends. In particular, Boning et al. (2016) extrapolated their FWF time series from 2010 to 2020 to investigate the longer term impacts on the AMOC. They note that the background variability in FWF is typically larger than the anomaly from the GrIS until 2020, when it reaches about 7,000 km^3^, which is close to 30% of the internal variability. It is interesting to note that the *FWF_CA_* estimated here is already close to this value by 2016 as a result of extending the length of the record and including glaciers and ice caps outside of Greenland. Not all of this, however, reaches areas of deep convection (Figure 4) (Boning et al., 2016; Gillard et al., 2016) and the partition between solid and liquid phases may be important in determining the proportion of the FWF that might interfere with convection and stratification. Nonetheless, using the *FWF_CA_* rate for the last decade (373 km^3^/yr) suggests that the anomaly will exceed 7,000 km^3^ by 2018 and 10,000 km^3^ by 2028.

**Figure 6:**
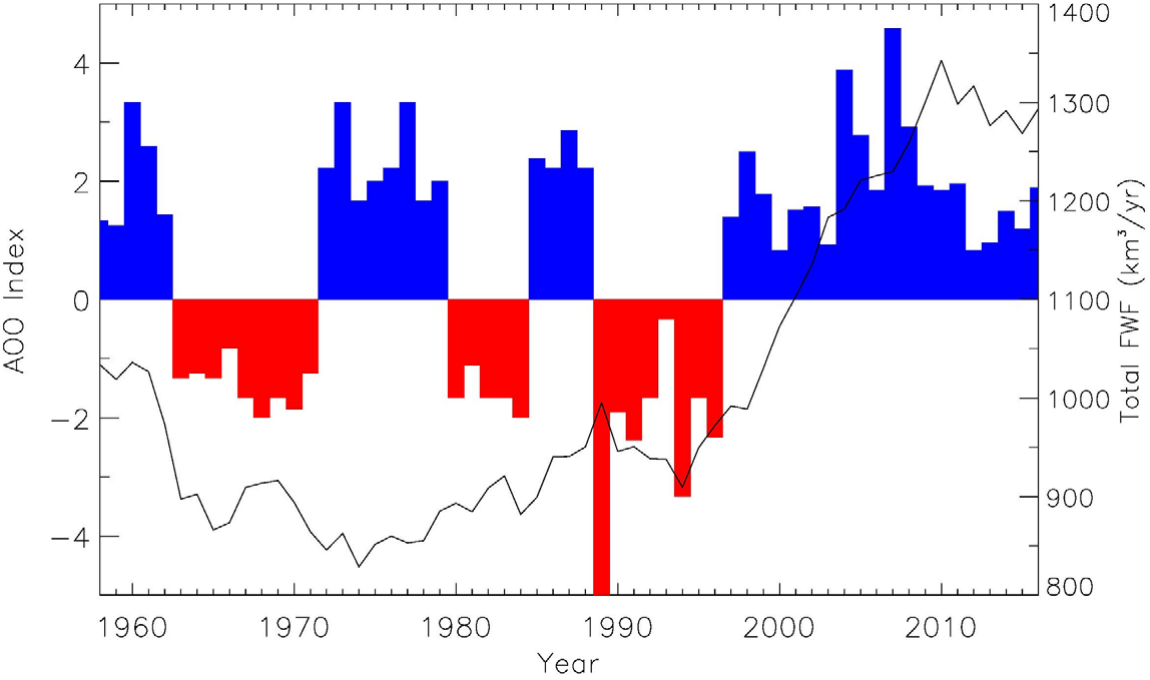
The Arctic Ocean Oscillation (updated from Proshutinsky et al., 2015) plotted alongside the total FWF anomaly from this study.

Other studies, taking a different approach, have also suggested that the enhanced melting from Arctic land ice is already influencing overturning strength and convection (Rahmstorf et al., 2015; Robson et al., 2016; Yang et al., 2016). In one such study, the onset of enhanced melting over Greenland was found to be roughly coincident with a shift in the Arctic Ocean Oscillation (AOO) index to a strongly positive phase (Figure 6) that has persisted since (Proshutinsky et al., 2015). This index, it has been argued, is more relevant to Arctic circulation regimes than other climate indices such as the North Atlantic Oscilliation (Proshutinsky et al., 2015). The AOO is a measure of the strength and dominant direction of atmospheric circulation over the Arctic that influences, for example, sea ice drift and Ekman transport. From 1946 to 1996 it showed a 5–7 year periodicity (Figure 6). Since 1997, however, it has experienced a prolonged positive phase that, based on a box model study, has been linked to enhanced FWF from Greenland (Proshutinsky et al., 2015). It has also been suggested that the upper ocean cooling in the North Atlantic, noted since 2005, may, in part, be due to increased FWF from the GrIS (Robson et al., 2016).

**Figure 7:**
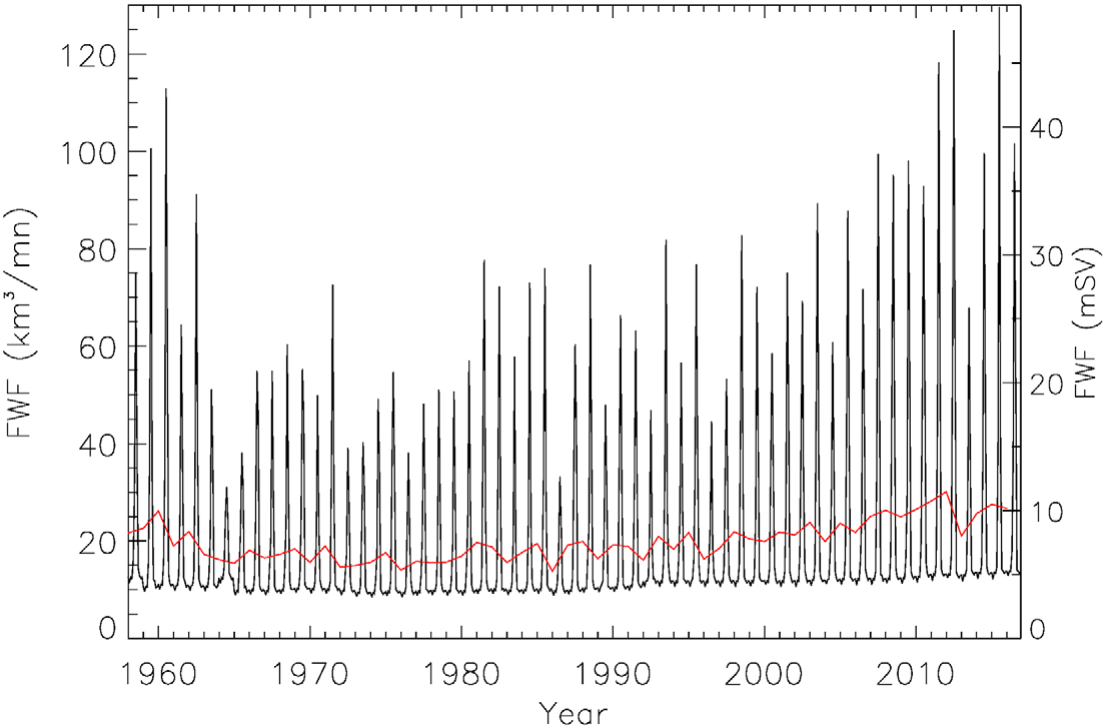
Monthly FWF for the Baffin Bay basin as solid black line. The units are km^3^/month on the left‐hand axis and mSv on the right‐hand axis. The annual flux is shown in red for comparison using the same units on the RHS.

To date, however, no freshwater forcing experiments have been undertaken with our new times series and, as a consequence, the potential oceanographic impacts remain somewhat speculative. We aim to address this in a companion paper, in preparation, where the FWF anomalies presented here will be used to force an ocean GCM with one objective being to update the study of Boning et al. (2016), and similar experiments, with actual FWF anomalies as opposed to extrapolated values. Nonetheless, a number of studies have considered the potential impact of the well‐documented accumulation of freshwater in Baffin Bay, (de la Guardia et al., 2015; Grivault et al., 2017). Figures 4 and 5 suggest a significant positive anomaly in this sector and this is supported by satellite observations (Carret et al., 2017) and ocean reanalysis (Zuo et al., 2015). For this basin, it has been suggested that there could be a potential positive feedback caused by the halosteric sea surface height anomaly (due to freshening) resulting in warmer water reaching marine‐terminating glaciers, which, in turn, would increase the FWF anomaly (de la Guardia et al., 2015). The FWF anomaly may, therefore, not only affect the regional hydrography but also the ice sheet proximal to Baffin Bay.

it is important to note that the FWF is not delivered uniformly in space or time. Significant surface runoff only occurs during summer months, predominantly, from June to August: a fact that has been incorporated in some recent freshwater forcing experiments (e.g., Dukhovskoy et al., 2016; Grivault et al., 2017). The seasonality in the FWF is important in determining its role on ocean stratification, biological productivity and fjord circulation (Straneo & Heimbach, 2013). The seasonality and its variability for the Baffin Bay sector is illustrated in Figure 7. Other sectors show a similar seasonal cycle and variability. The relatively constant wintertime FWF of about 10 km^3^/month is due to D and increases gradually from the early 1990s. Evidence from ice mélange in two Greenland fjords suggests that the solid ice discharge provides a continuous and dominant FWF to the fjord, except during high summer when runoff can exceed submarine melt rates (Enderlin et al., 2016). Peak FWF occurs around July and is typically an order of magnitude larger than the winter time values and about five times larger than the annual mean. In ocean GCM hosing experiments, this seasonality may be important in determining the fate of the freshwater and its influence on stratification (Marsh et al., 2010; Straneo & Heimbach, 2013) as well as the delivery of bioavailable nutrients to coastal waters (Hawkings et al., 2016).

Not only is the FWF distributed unevenly geographically but also vertically within the water column. In the case of marine terminating glaciers some proportion of the surface runoff reaches the bed and is released at the base of the glacier (Sole et al., 2011), which can be many hundreds of meters below sea level. For Jakobshavn Isbrae in southwest Greenland, the grounding line depth in 2016 was at 1,100 m below sea level, for example (An et al., 2017). While this may be an end member, many marine terminating outlet glaciers in Greenland lie in deeply incised troughs that are several hundred meters below sea level (Morlighem et al., 2014, 2017). In addition, the icebergs that calve from theses glaciers will be delivering freshwater across a range of depths. Of greater significance for experiments attempting to use observed FWF estimates, as opposed to idealized values, is that the icebergs are not stationary and drift due to both oceanic and wind forcing (Marsh et al., 2015). Icebergs calving along the east Greenland coast tend to drift northward through the Fram Strait and into the Arctic Ocean, while those from the west coast are carried along the West Greenland Current into Baffin Bay and southward along the Labrador Current (Marsh et al., 2015). As mentioned earlier, it is incorrect to assume, therefore, that the entire land ice FWF will reach key areas of overturning circulation or that it is uniformly distributed at the surface at a constant rate through the year. Future ocean GCM experiments should include this variability in delivery if they aim to understand the fate and role of land ice FWF on ocean processes.

In this paper we derive a new estimate of freshwater fluxes from terrestrial land ice that contributes to the Arctic and North Atlantic Oceans. Compared to a previous assessment for the Greenland Ice Sheet alone, the cumulative freshwater anomaly is about double. This is partly due to the addition of glaciers and ice caps outside of Greenland but also to the additional years considered here, which extends the time series to the end of 2016. Due to limitations in data coverage and model domain, we did not include FWF estimates for the Russian Arctic and discharge changes in Svalbard. We estimate that these omissions represent about 5% of the FWF anomaly we obtained, which is below the uncertainties in the time series. The total cumulative FWF anomaly is close to reaching a value where it will be comparable to the interannual variability in FWF driven by other processes in the climate system (Boning et al., 2016). In a companion paper (part II), we consider the hydrographic and climate‐related consequences of the freshwater trends presented here by forcing and ocean GCM with the FWF anomalies using tracers (Boning et al., 2016; Dukhovskoy et al., 2016). The data set described here is available as a netcdf file that contains the land/ice/ocean mask and ocean basin definitions used (Figure 4) along with separate fields for tundra, ice runoff and solid ice discharge.
